# Safety profile of *colocasia esculenta* tuber extracts in benign prostate hyperplasia

**DOI:** 10.1186/s12906-023-04018-4

**Published:** 2023-06-08

**Authors:** Deusdedit Tusubira, Patrick M. Aja, Jonasi Munezero, Frank Ssedyabane, Nathim Namale, Josiah E. Ifie, Peter C. Agu, Clement O. Ajayi, Joash Okoboi

**Affiliations:** 1 Department of Biochemistry, Faculty of Medicine, Mbarara University of Science and Technology, Mbarara, Uganda; 2 Department of Biochemistry, Faculty of Sciences, Ebonyi State University, Abakaliki, Nigeria; 3 Department of Medical Biochemistry, Kampala International University, Kampala, Uganda; 4 Medical Laboratory Science, Mbarara University of science and Technology, Mbarara, Uganda; 5 Faculty of Medicine, Department of Pharmacy, Mbarara University of Science and Technology, Mbarara, Uganda; 6 Department of Biochemistry, Soroti University, Soroti, Uganda

**Keywords:** Neutraceutical, Benign prostate cancer, Coco yam

## Abstract

**Introduction:**

This study was motivated by the increasing global incidence of benign prostatic hyperplasia (BPH) and the promising potential of nutraceuticals as complementary therapies in ameliorating its burden. We report the safety profile of *C. esculenta* tuber extracts, a novel nutraceutical in benign prostate hyperplasia in a rat model.

**Methods:**

In this study, forty-five male albino rats were randomly assigned to 9 groups of 5 rats each. Group 1 (normal control) received olive oil and normal saline. Group 2 (BPH untreated group) received 3 mg/kg of testosterone propionate (TP) and normal saline, and group 3 (positive control) received 3 mg/kg of TP and 5 mg/kg of finasteride. Treatment groups 4, 5, 6, 7, 8, and 9 received 3 mg/kg of TP and a middle dose (200 mg/kg) of LD50 of ethanol crude tuber extract of *C. esculenta* (ECTECE) or hexane, dichloromethane, butanone, ethyl acetate and aqueous fractions of ECTECE respectively for a period of 28 days.

**Results:**

The negative controls showed a significant (p < 0.05) increase in mean relative prostate weight (approximately 5 times) as well as a reduction in relative testes weight (approximately 1.4 times less). There was no significant (p > 0.05) difference in the mean relative weights of most vital organs: liver, kidneys, and heart. This was also observed in hematological parameters: RBC, hemoglobin, HCT, MCV, MCH, MCHC, and platelets counts. In general, we note that the effects of the well-established drug finasteride on the biochemical parameters and histology of selected organs are comparable to those of *C. esculenta* fractions.

**Conclusion:**

This study demonstrates that C. esculenta tuber extracts provide potentially safe nutraceutical if applied in the management of benign prostate hyperplasia based on a rat model.

## Introduction

The increasing trend of life expectancies across the world [1] has been accompanied by increasing prevalence and incidence of benign prostate hyperplasia (BPH) [2–4]. Benign prostate hyperplasia as defined histologically by an overgrowth of prostate tissue [5], is among the commonest noncancerous form of abnormal prostate cell growth affecting older men globally [6–8]. In men over 50 years of age, global prevalence of BPH ranges between 20 and 62% [4, 9–11]. As the prostate enlarges, it constricts the urethra leading to symptoms which usually result into BPH associated lower urinary tract symptoms (LUTS) [2, 8, 12, 13]. In Uganda, the prevalence rates for LUTS in men above 55 years of age were estimated to be as high as 40.5% in a study published in 2018 [14]. Apart from the cost [10, 15] and health systems challenges, poor treatment outcomes in BPH have been linked to real and perceived side effects of commonly used pharmacological regimens [15–18].

Conventional pharmacological management of BPH usually involves use of alpha-blockers comprising alpha-adrenoceptor antagonists, or 5-alpha-reductase inhibitor or a combination of both [15, 17, 19]. These treatment options are associated with severe side effects including but not limited to organ toxicity and sexual dysfunction [20–22]. Moreover most of these patients are older adults with multiple co-morbidities and medications [23]. There is an urgent need for additional research into novel approaches to treatment of BPH which could involve incorporation of alternative or complementary therapies [8].

There are vast quantities of unexplored novel phytotherapeutic agents from commonly consumed foods that could provide better options for management and/or prevention of BPH [24, 25]. Such options are usually associated with fewer side effects due to long history of traditional use as foods [24] although more data is required to get a better understanding of their safety profiles [26]. *C*. *esculenta* (English name cocoyam or Taro, local name in Buganda region of Uganda, ‘*Obukopa*’ and in Igbo (*Onitsha*) Nigeria is among the neglected but important foods across the African continent [27–29]traditionally used in the management of BPH especially in West African countries [29–31]. Medicinal properties of *C. esculenta in-vitro* and *in-vivo* have previously been reviewed by Prajapati et al. (2011) [29]. Moreover, studies by Brown et al. (2005) suggest that *C.esculenta* has novel tumor specific anti-cancer activities on rat YYT colon cancer cell line [32]. Furthermore, using animal models, Kalariya et al.(2015) [33] reported that (25 and 50 mg/kg, i.p.) of hydroalcoholic extract of leaves of *C. esculenta* decreases obsessive-compulsive disorder in mice. Some studies have also demonstrated the anti-diabetic properties of *C.esculenta* tuber and leaves in rat model [34–37].

**Fig. 1:**
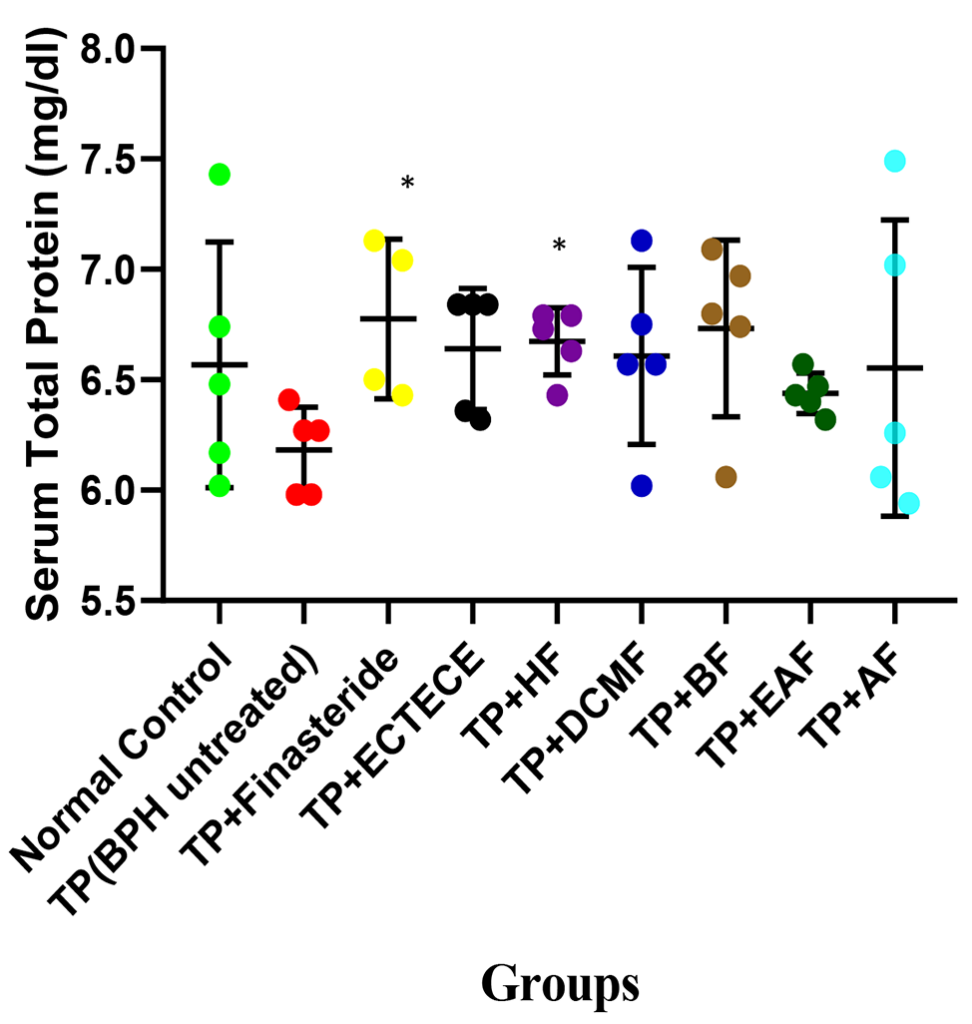
Effect of ethanol crude Tuber extract of *Colocasia esculenta* and Fractions on Serum Total Protein concentration in Testosterone propionate induced benign prostate hyperplasic Rats. Data are shown as mean ± S.D (n = 5). Mean values of different groups were compared with the control using Dunnet ANOVA with significantly difference at P < 0.05 (indicated by *). Testosterone propionate (TP), Ethanol crude Tuber extract of *Colocasia esculenta* (ECTECE), Hexane fraction(HF), Dichloromethane fraction( DCMF), Butanone fraction(BF), Ethyl acetate fraction(EAF) and Aqueous Fraction (AF)

Previous studies by Eleazu and others [38] based on a rat animal model demonstrated that *C. esculenta* contains anti-inflammatory agents that may remediate BPH associated inflammation. Studies by Nzebang et al.(2018) [39] suggest that the aqueous extract from *C. esculenta* leaves infected by *Phytophthora colocsiae* would be no major health risk with estimated LD_50_ of more than 4000 mg/kg in rats Although our previous studies have demonstrated that the ethanol crude extract of *C. esculenta* has the capacity to reduce prostate weight, total protein as well as serum concentration of prostate specific antigen [40] there was need to further explore the safety profile and associated biochemical mechanisms. This study was therefore designed to investigate the safety profile of *C. esculenta* in crude and semi purified form for the management and / or prevention of BPH.

**Fig. 2:**
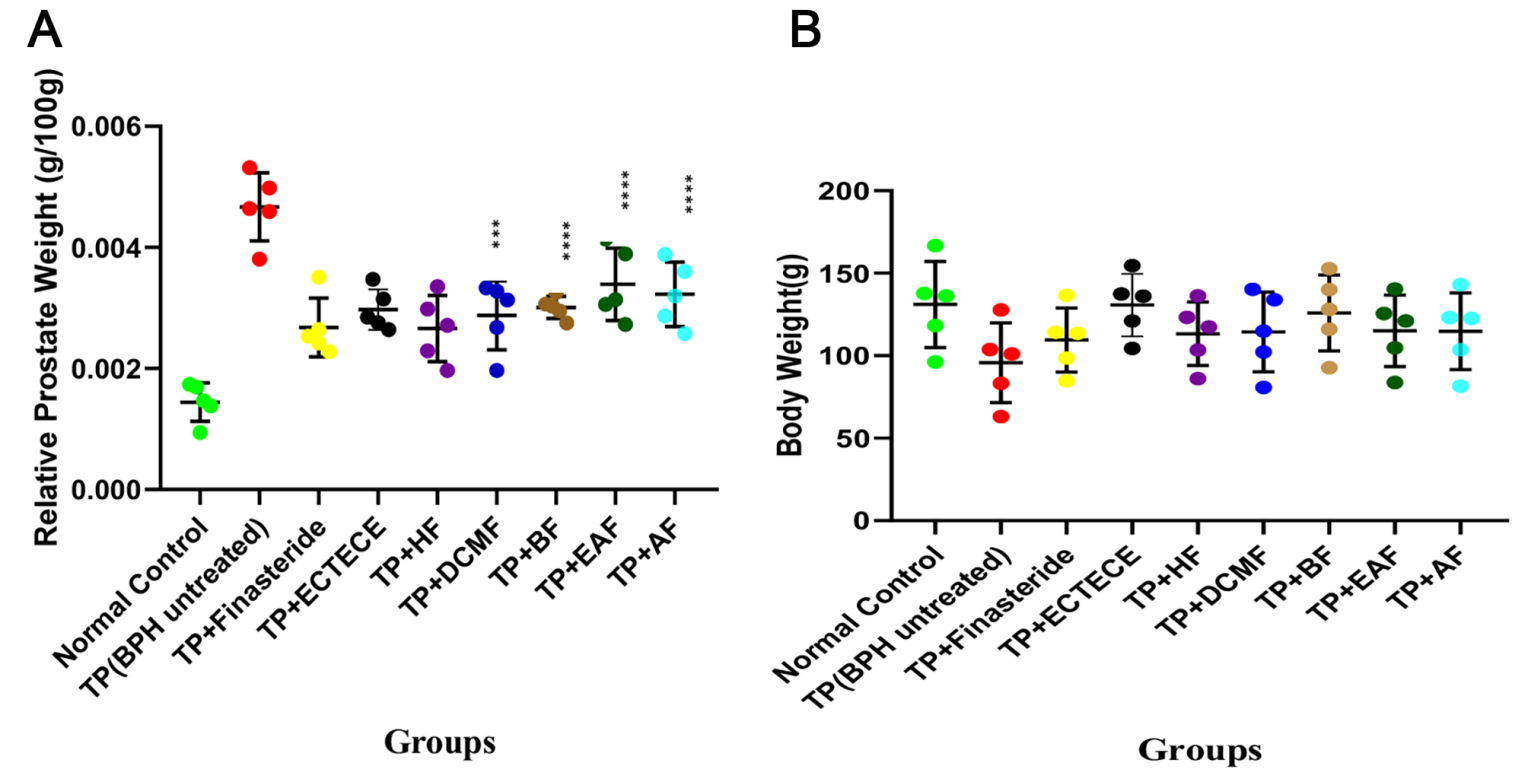
Effect of ethanol crude Tuber extract of *C. esculenta* and Fractions on Relative Prostrate Weight in Testosterone propionate induced benign prostate hyperplasic Rats. Relative prostate weight was calculated by dividing the weight of the prostate of the animal divided by the body weight of the animal. Mean values of different groups were compared with the control using Dunnet ANOVA with significantly difference at P < 0.0002 (****) and P < 0.002 (***). Testosterone propionate (TP), ethanol crude Tuber extract of *C. esculenta* (ECTECE), hexane fraction (HF), dichloromethane fraction (DCMF), butanone fraction (BF), ethyl acetate fraction (EAF) and aqueous fraction (AF)

## Materials and methods

**Collection and Identification of Plant Materials:** Mature (6–8 months old) fresh tubers of *C. esculenta* were collected with permission from the selected farmers and local authorities including agricultural extensions officer as guided by the Office of Research, Innovation and Institutional Ethics Committee of Ebonyi state university, Nigeria (EBSU/BCH/ET/21/001). This was done in accordance with guidelines from the Nigerian Federal Environmental Protection Agency and the International Union for Conservation of Nature (IUCN) policy on Research Involving Species at Risk of Extinction. This was followed by authentication by a taxonomist, Professor S.O. Onyekwelu at the department of Applied Biology, Ebonyi state University (*Colocasia esculenta (L) Schott*, Family *Araceae*, common name: Cocoyam Local name; *Ede ofe*, voucher number: EBSU-H-206, Department of applied biology herbarium, Ebonyi State university, Nigeria ; Herbarium curator, Mr. Nwanko Onyebuchi Ephraim.

**Fig. 3:**
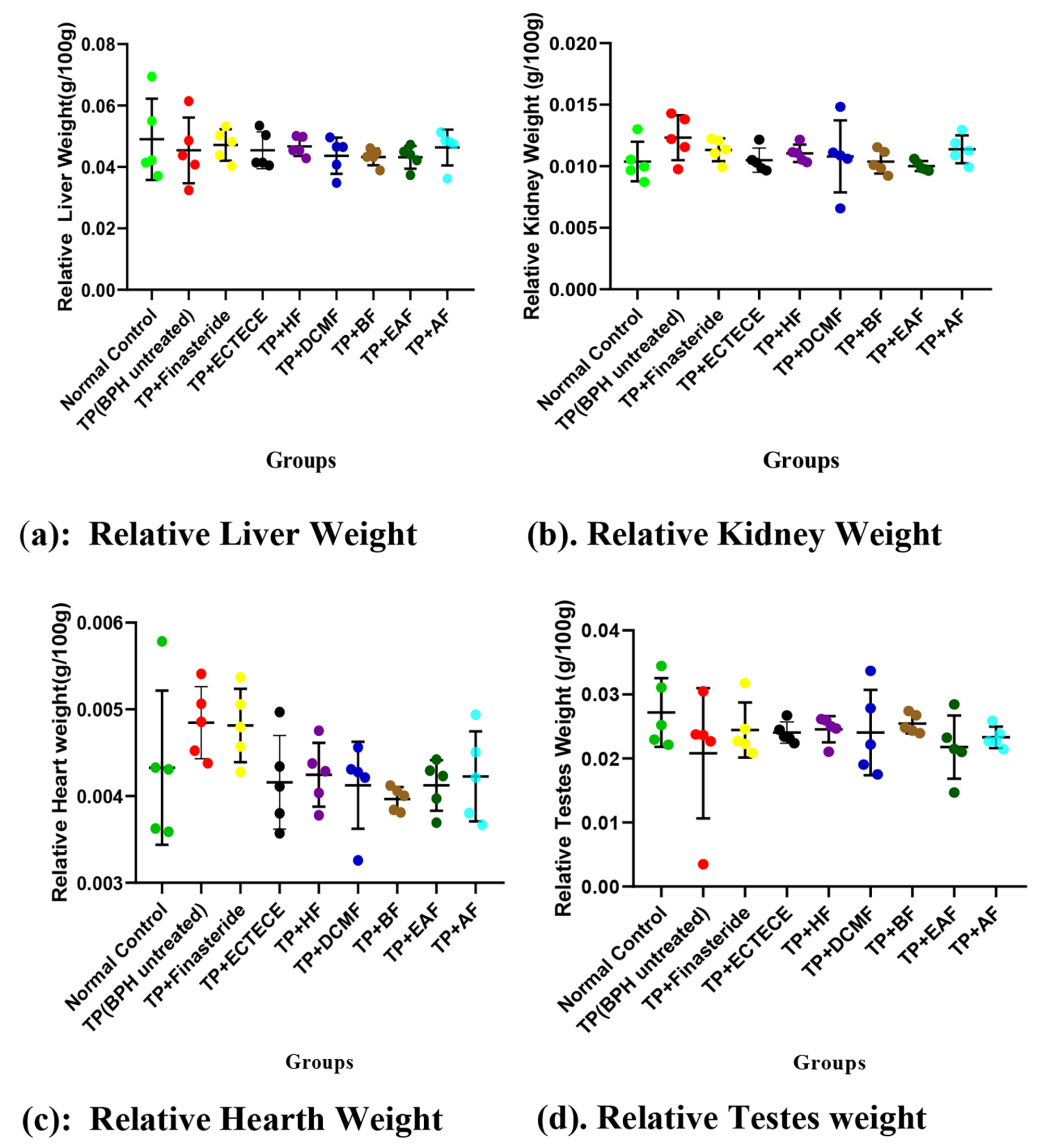
(a-d): Effect of ethanol crude Tuber extract and Fractions of *C. esculenta* on Relative Organs weights in Testosterone propionate induced benign prostate hyperplasic Rats. Data are shown as mean ± S.D (n = 5). Mean values of different groups were compared with the control using Dunnet ANOVA with significantly difference at P < 0.05 although none of them was significant. Testosterone propionate (TP), Ethanol crude Tuber extract of *C. esculenta* (ECTECE), Hexane fraction (HF), Dichloromethane fraction (DCMF), Butanone fraction (BF), Ethyl acetate fraction (EAF) and Aqueous Fraction (AF).

### Preparation of Plant materials:

*C. esculenta* tubers were washed, boiled, peeled, sliced into chips, air-dried to a constant weight at a room temperature and processed into flour.

### Extraction of plant materials

The powdered tuber 1280 g of *C. esculenta* were extracted with 8 L of 50% ethanol (Emsure®) overnight in a big stopper bottle with occasional stirring at room temperature. It was then sieved using muslin cloth. The filtrates were air dried for 24 h to get the ethanol (crude) extracts.

### Purification by Solvent extraction using partition coefficient:

The crude natural ethanol product was extracted with solvents of increasing polarity, first, hexane (Blulux), dichloro methane (UNILAB), ethyl acetate (UNILAB) and butanol (AnalaR®) which depended on the chemical and physical nature of the target compounds.

**Fig. 4:**
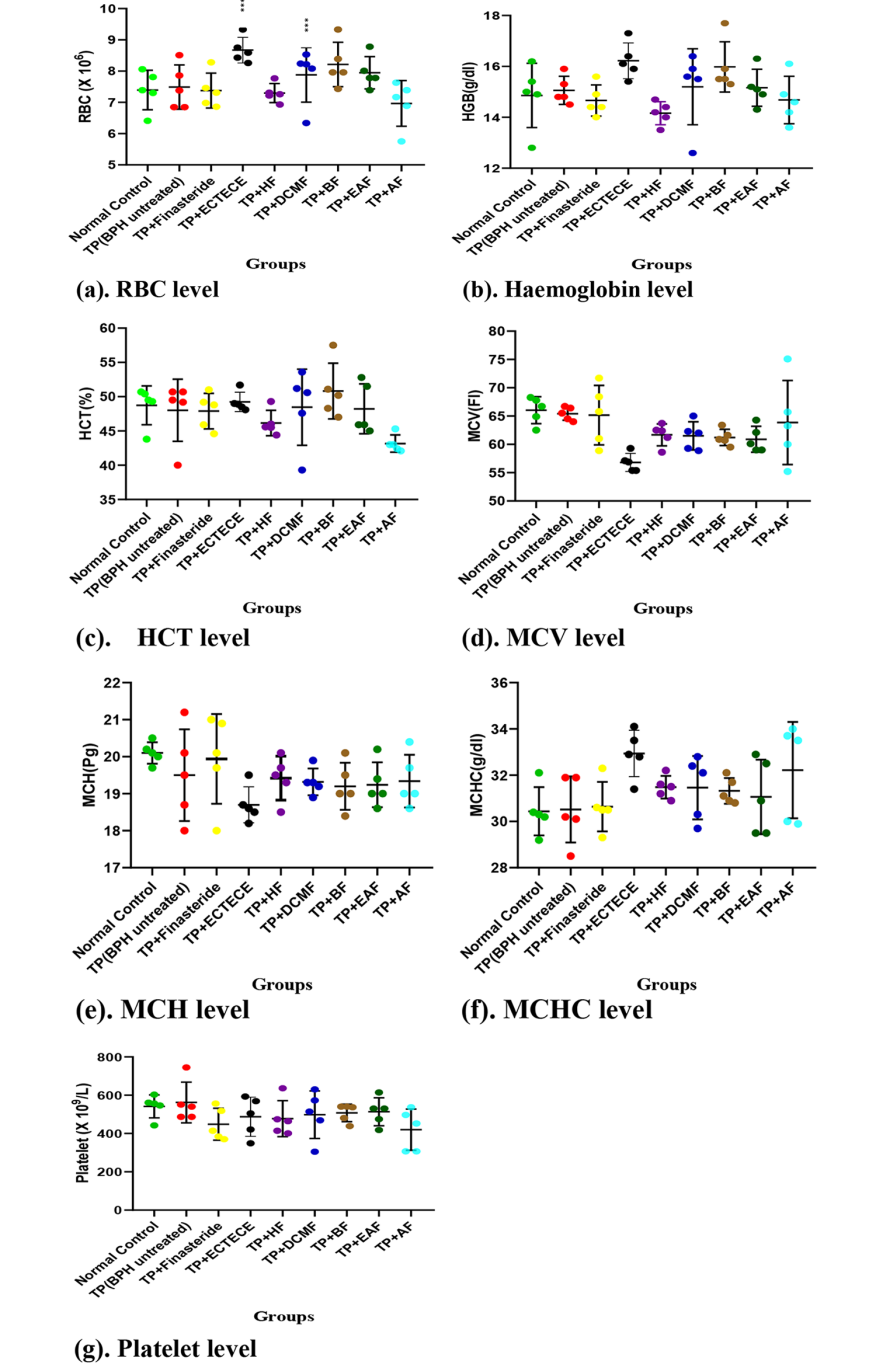
(a-g): Effect of ethanol crude Tuber extract of *Colocasia esculenta* and Fractions on Haematological indices in Testosterone propionate induced benign prostate hyperplasic Rats. Mean values of different groups were compared with the control using Dunnet ANOVA with significantly difference at P < 0.003 (***) and P < 0.001 ( ****). Testosterone propionate (TP), Ethanol crude Tuber extract of *Colocasia esculenta* (ECTECE), Hexane fraction(HF), Dichloromethane fraction( DCMF), Butanone fraction(BF), Ethyl acetate fraction(EAF) and Aqueous Fraction (AF)

This study was done under the supervision and approval by the Office of Research, Innovation and Institutional Ethics Committee of Ebonyi state university, Nigeria (EBSU/BCH/ET/21/001). All procedures for animal studies were performed following guidelines and legislations consistent with the National Institute of Health Guide for the Care and Use of Laboratory Animals (NIH Publications No. 80-23, revised in 1996) [33] as well as the National guidelines for the use of laboratory animals for research and teaching based on the principles of 3Rs, reduce refine or replace.

**Fig. 5:**
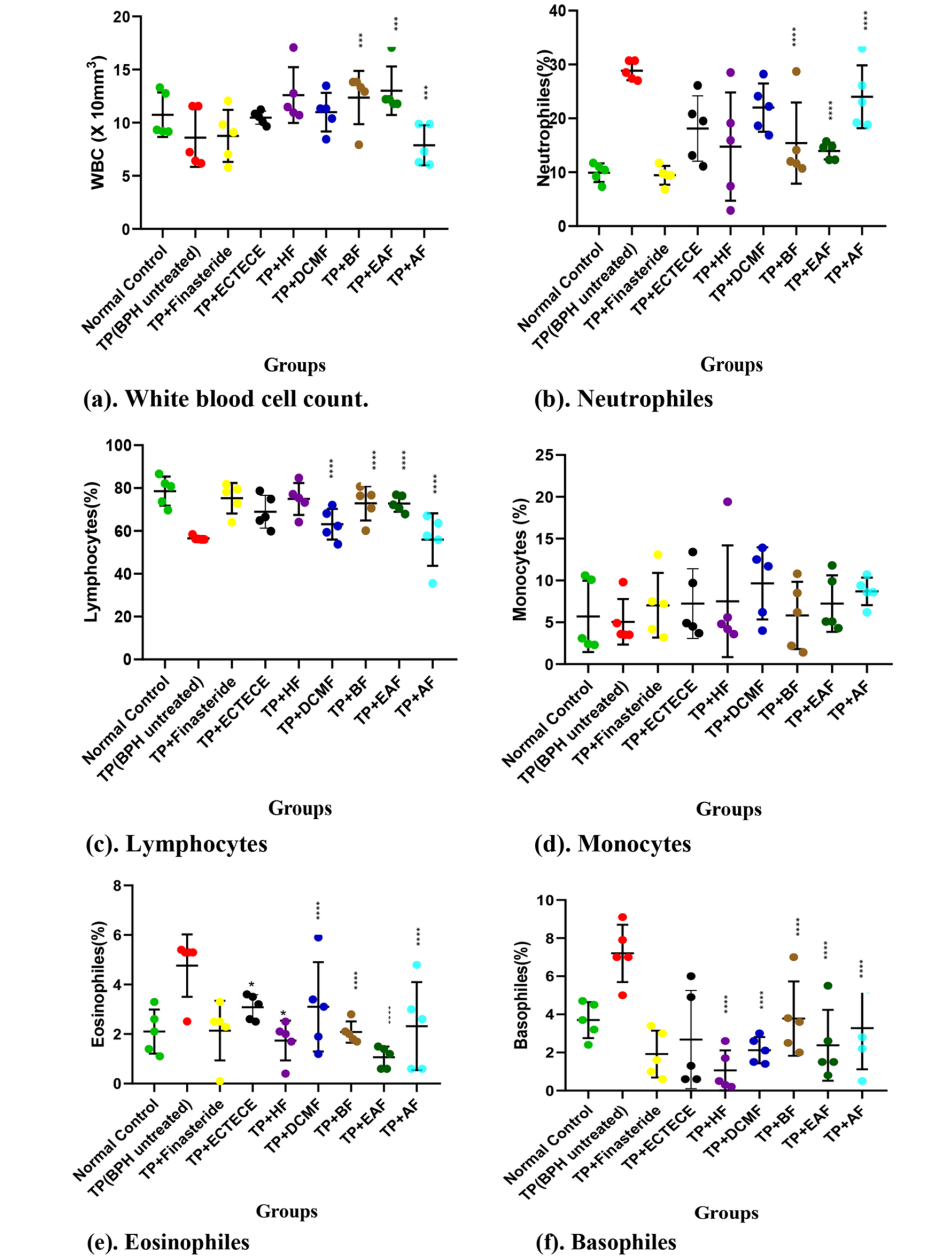
(a-f): Effect of ethanol crude Tuber extract of Colocasia esculenta and Fractions on WBC level and differential counts in Testosterone propionate induced benign prostate hyperplasic Rats. Data are shown as mean ? S.D (n=5). Mean values of different groups were compared with the control using Dunnet ANOVA with significantly difference at P<0.05. Testosterone propionate (TP), Ethanol crude Tuber extract of Colocasia esculenta (ECTECE), Hexane fraction(HF), Dichloromethane fraction( DCMF), Butanone fraction(BF), Ethyl acetate fraction(EAF) and Aqueous Fraction (AF), BPH( Benign prostate hyperplasia)

### Laboratory animals

The study used 45 male Wistar albino rats of about 6 weeks old which were sourced from the Animal House of Ebonyi state university, Nigeria. They were kept in stainless cages (size: 16 × 9 = 144inches) in a well-ventilated animal house. They were acclimatized for seven days under good laboratory conditions (12 h light/dark cycle; room temperature) before the start of the experiments. All animals were allowed free access to standard rodent chow and water *ad libitum*. All animals were humanely sacrificed using halothane.

**Fig. 6:**
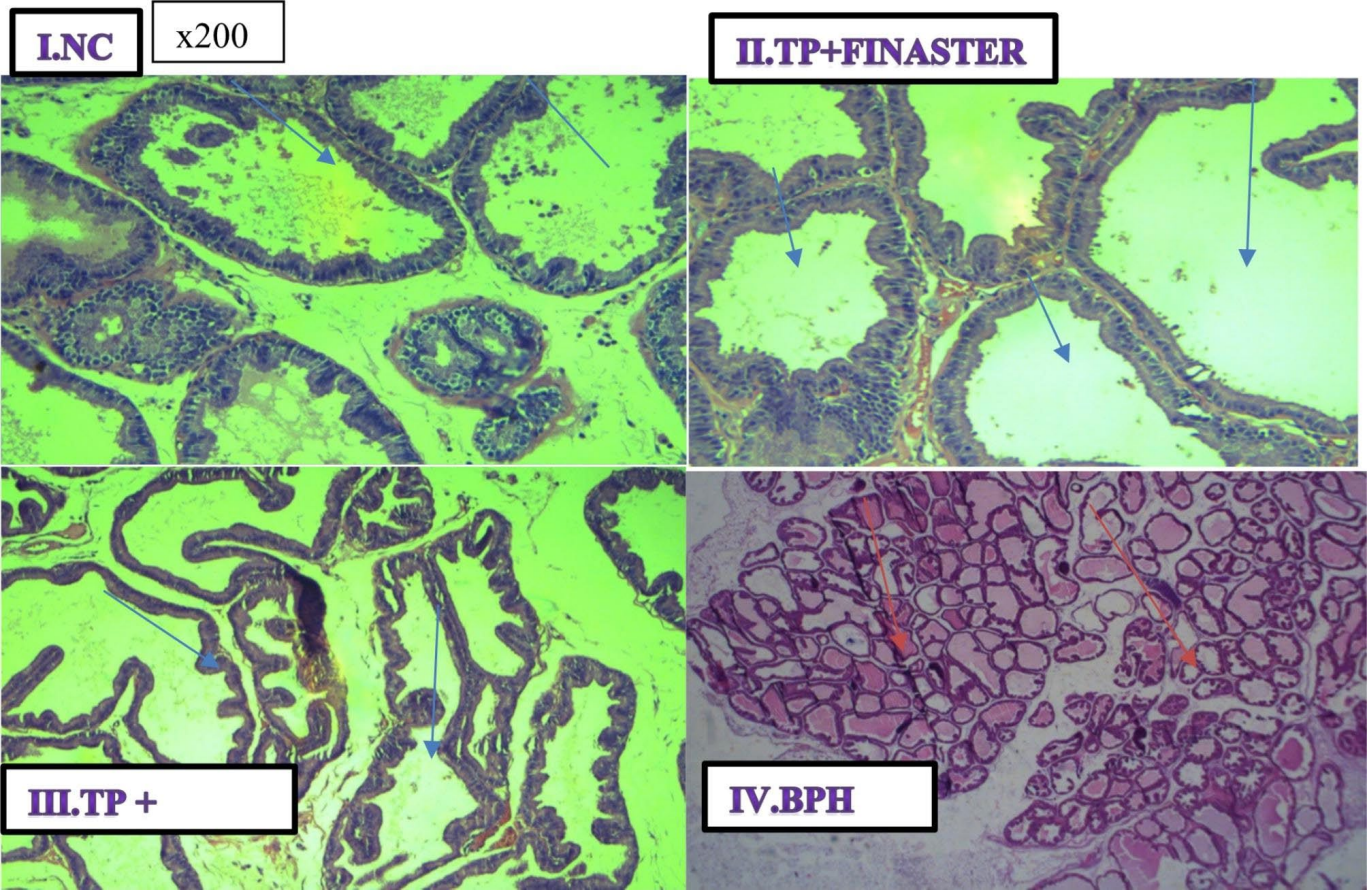
Haematoxylin and Eosin stain on histological sections of prostate gland representative image from three rats selected from each of the group. (I) (NC) Normal control group (magnification, x200); (II) (TP + Finasteride) Positive control group (magnification, x200); (III) TP + ECTECE (magnification, x200); iv. (BPH) Negative control group (magnification, x200). Blue arrows show the prostate glands with no proliferating prostate cells while the red arrows show proliferating prostate cell, BPH- Benign prostatic hyperplasia, ethanol crude tuber extract of *C. esculenta*

### Induction of benign prostatic hyperplasia

We induced Benign Prostatic Hyperplasia in rats using testosterone propionate (TP) [34, 35]. The dose for induction was formulated as 3 mg/kg body weight and it was given by subcutaneous injection every day for 28 days. We prepared stock by dissolving 25 mg of TP in 8.33ml olive oil. Three rats each from the groups were randomly selected for confirmation of BPH before treatment. Prostate Specific Antigen (PSA) concentrations of the rats were determined using the method by Stowell et al. [41].

### Grouping of animals

The rats were grouped as follows, with five rats in each group: Rats in group 1 (Normal control) received subcutaneously 1ml of olive oil. BPH was induced in groups 2–9 with 3 mg/kg TP subcutaneously. Group 2 (BPH untreated group) was not treated. Group 3 (Finasteride group) had rats which were treated with 5 mg/kg finasteride. The middle dose of 200 mg/kg was used for this study as reported by Eleazu *et al. (*2021). Group 4 had rats which were treated with 200 mg/kg body weight (b.w) of ethanol crude tuber extract of *C. esculenta* (ECTECE). Group 5 had rats that were treated with 200 mg/kg b.w of n-hexane fraction (HF). Group 6 had rats that were treated with 200 mg/kg b.w of dichloromethane fraction (DCMF). Group 7 had rats that were treated with 200 mg/kg b.w of ethyl acetate fraction (EAF). Group 8 had rats that were treated with 200 mg/kg b.w of butanone fraction (BF). And finally, group 9 had rats that were treated with 200 mg/kg b.w of aqueous fraction (AF). Oral administration of the extract or fractions or finasteride was done using oral gavage and all animal diets were provided *ad libitum*.

**Fig. 7:**
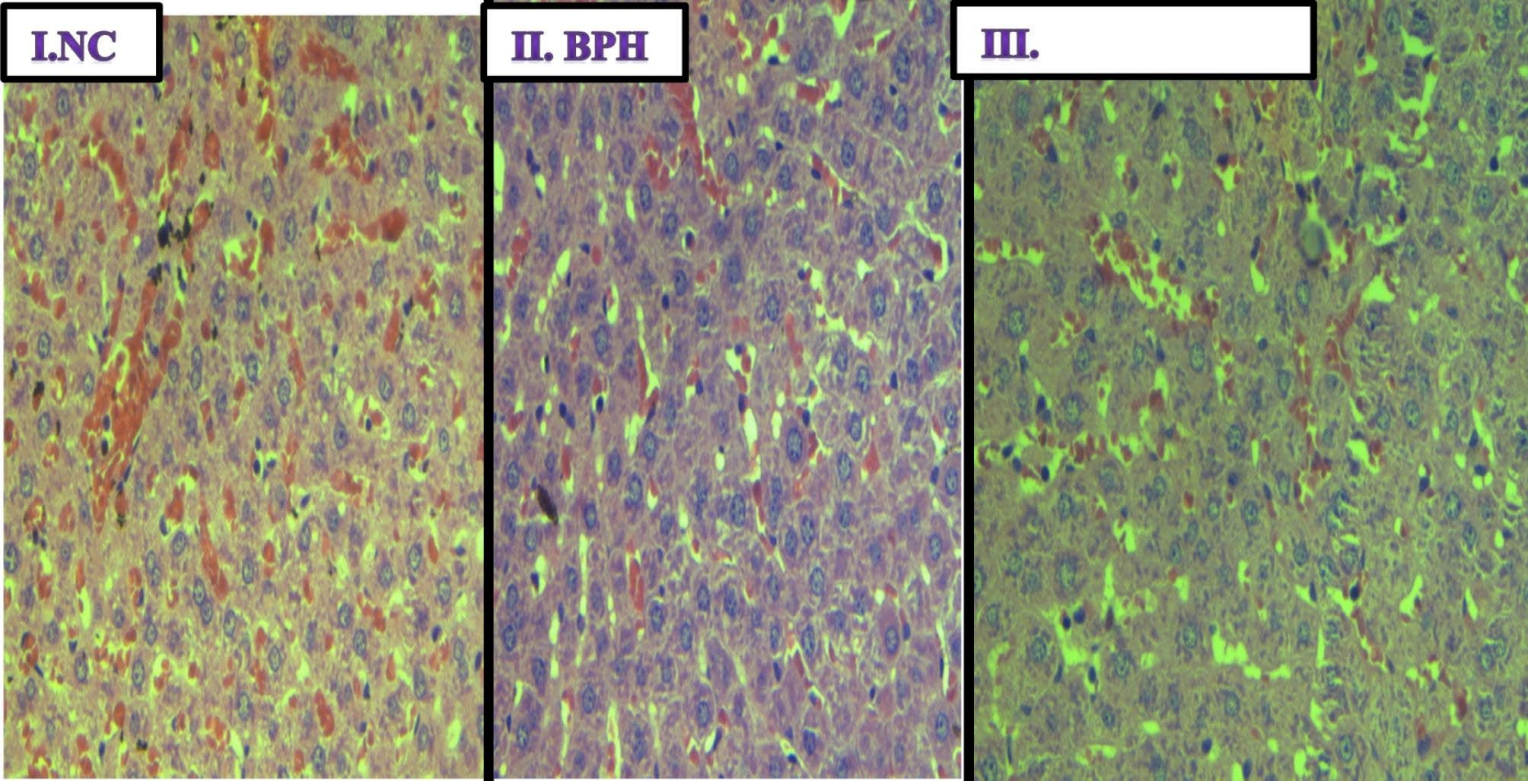
(I-III). Haematoxylin and Eosin stain on histological sections of the liver. (I) Normal control group (NC) (magnification, x200); (II) BPH untreated group (magnification, x200); and (III) TP + ECTECE group (magnification, x200). BPH- Benign prostatic hyperplasia, ethanol crude tuber extract of *C. esculenta*

### Laboratory analysis

After 28 days, the rats were fasted overnight, sacrificed after being anesthetized with halothane and dissected to collect vital organs which included liver, kidney, heart as well as prostate and testes. All organs were weighed and immediately placed in 10% neutral buffered formalin for fixation until histopathological examination. We also collected blood samples from animals by cardiac puncture, using 5ml syringes into EDTA vacutainers for complete blood count. Anticoagulated blood samples were stored at 2 to 8^0^c for two days after which a complete blood count was performed.

Total protein was determined using the method in Tietz [42]. The body weights, prostrate, liver, kidney, heart and testes weights of the rats were recorded on a daily basis, using an electronic weighing balance (Model Scout Pro, Ohaus Corporation, USA), and were calculated as follow: Relative prostrate weight (g/100 g) = Total Prostrate weight/Final body weight x100; Relative liver weight (g/100 g) = Total liver weight/Final body weight × 100; Relative kidney weight (g/100 g) = Total kidney weight/ Final body weight × 100; Relative heart weight (g/100 g) = Total heart weight/ Final body weight × 100 and Relative Testes weight (g/100 g) = Total Testes weight/ Final body weight × 100.

For complete blood count, we used a five-part differential fully automated analyser, Sysmex XN550, Japan. Sample tubes with caps closed were placed in sample adapters. We then used the start/stop switch to execute automatic sample mixing, aspiration, analysis and printing of results.

**Fig. 8:**
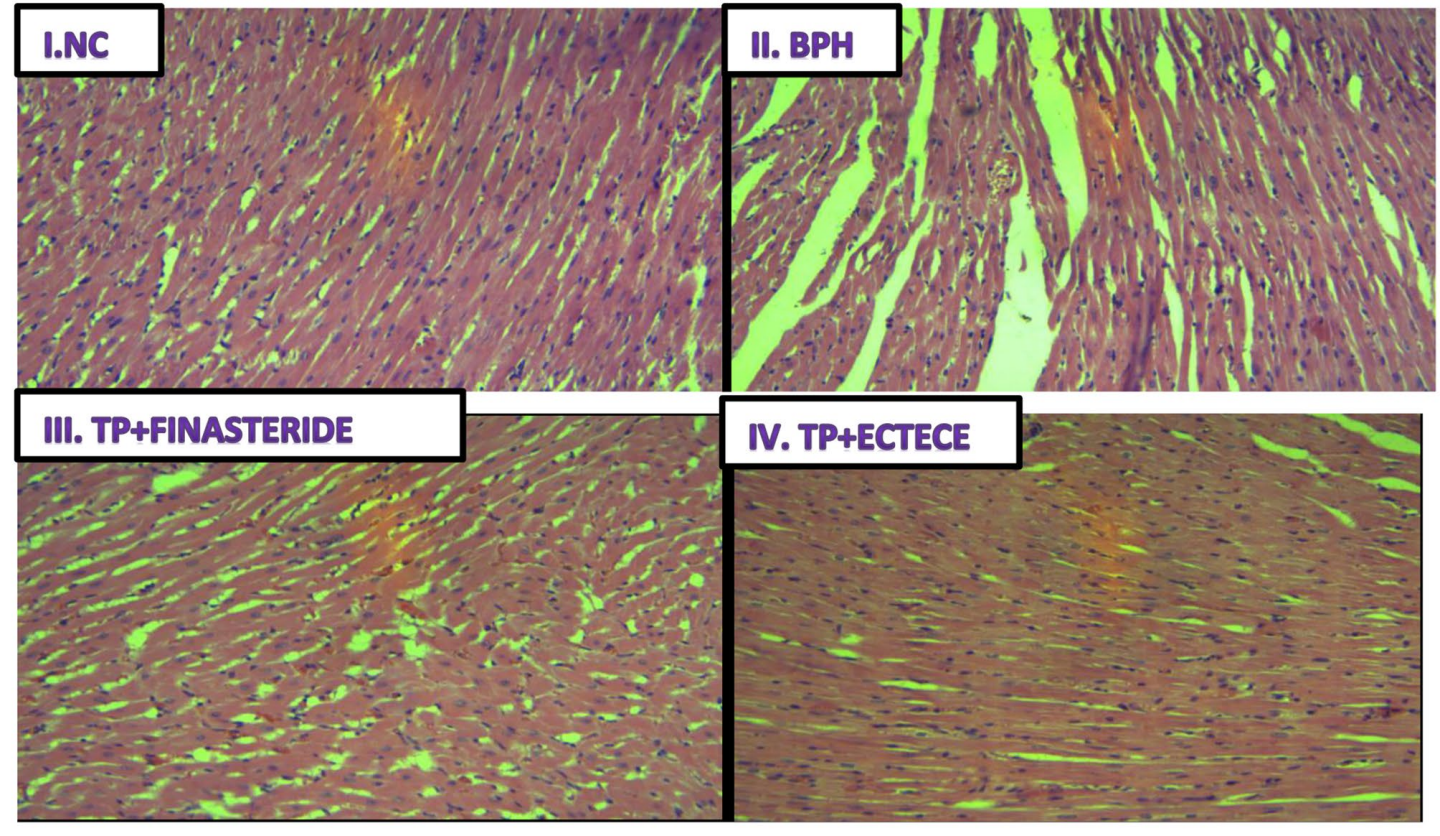
(I-IV) showed no demonstrable hyperplasia, atrophy or hypertrophy of heart tissue in the normal control group (NC), neither was it demonstrated in the BPH untreated group nor TP + Finasteride groups. The same was observed in TP + ECTECE group

### Histopathological examination

The histopathological examination of the organs was done using standard procedures [42]. Tissues were grossed and small pieces were cut out and placed in tissue cassettes for processing. Tissue processing included completion of fixation in 10% neutral buffered formalin, dehydration in increasing concentrations of ethanol, clearing in two changes of xylene and impregnation with molten paraffin wax. Tissues were then embedded using Tissue-Tek embedding moulds and sectioned using a microtome set at 5 μm. Tissue sections were stained using the Haematoxylin and Eosin (H and E) stain and later examined under a microscope. Three animals were selected from each group and taken for analysis. Technicians were blinded to the group. Each section was reviewed by two independent pathologists. Pictures were taken at x200 magnification. Representative images were selected with the expert guidance of the pathologist.

**Fig. 9:**
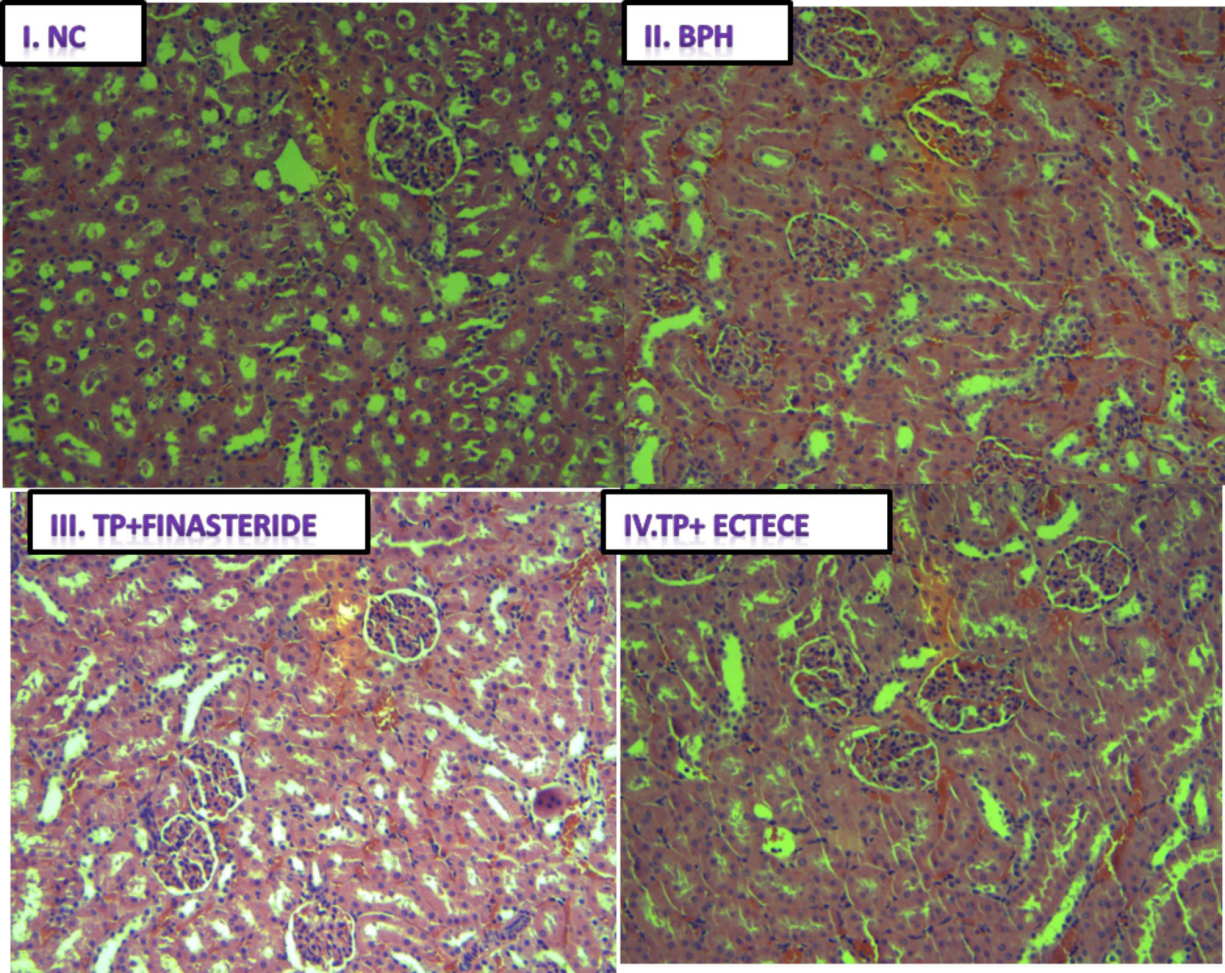
(I-IV). Haematoxylin and Eosin stain on histological sections of kidney tissue. (I) Normal control (NC) group (magnification, x200); (II) BPH control group (magnification, x200); (III) TP + Finasteride (magnification, x200) and (IV) TP + ECTECE (magnification, x200). BPH- Benign prostatic hyperplasia, ethanol crude tuber extract of *C. esculenta*

### Quality control

For the complete blood count, we used control blood and normal samples to monitor daily variation. We also employed the XN check levels 1, 2 and 3. In addition, we followed manufacturer’s instruction and standard operating procedures for equipment start-up, self-check, as well as sample preparation and loading.

For histopathology, we ensured use of standard operating procedures and all stains and reagents were used after filtration. Examination of slides was done by two qualified pathologists and in cases of a discrepancy, a third pathologist was involved.

### Statistical data analyses

Data were analyzed using Prism software (Graph-Pad Software; San Diego, CA) to determine statistical significance. Dunnet ANOVA test was used to compare the mean values of the individual groups and control at P < 0.005. The results are shown as mean ± SD of 5 rats per group.

## Results

### Effect of ethanol crude tuber extract of ***colocasia esculenta*** and fractions on serum prostate specific antigen (PSA) and total protein in testosterone propionate (TP) induced benign prostate hyperplasic (BPH) rats

Administration of TP in male Wistar albino rats significantly (p < 0.05) elevated serum PSA level in Table 1. Co-administration of TP and ethanol crude Tuber extract of *Colocasia esculenta* (ECTECE), hexane fraction (HF), dichloromethane fraction (DCMF), butanone fraction (BF), Ethyl acetate fraction (EAF) and aqueous fraction (AF) in male rats significantly (p < 0.05) reduced the level of serum PSA in comparison to the normal control group with no significant change (p > 0.05) in total protein concentration in the serum. Interestingly, significant (p < 0.05) reductions in the level of serum PSA were observed in all the fractions except butanone fraction when compared with the normal control group (Table 1).

**Table 1:** Effect of ethanol crude tuber extract of *C. esculenta* and Fractions on serum prostate specific antigen in TP induced BPH Rats

Groups Parameters	BPH untreated	TP(BPH untreated)	TP + Fin	TP + ECTECE	TP + HF	TP + DCMF	TP + BF	TP + EAF	TP + AF	P value
Serum PSA	0.182^a^ ± 0.01	0.209^b^± 0.03	0.008^c^± 0.00	0.081^d^± 0.05	0.056^e^± 0.03	0.145^e^± 0.07	0.219^ g^± 0.05	0.117^ h^± 0.04	0.097^i^± 0.07	0.0001

Data are shown as mean ± S.D (n = 5). Dunnet ANOVA test was used to compare the mean values of the individual groups and control at P < 0.005. Mean values with different alphabets are showed significantly difference at P < 0.05. NC (Normal Control), Testosterone propionate (TP), Fin( Finasteride), Ethanol crude Tuber extract of *C. esculenta* (ECTECE), Hexane fraction(HF), Dichloromethane fraction( DCMF), Butanone fraction(BF), Ethyl acetate fraction(EAF) and Aqueous Fraction (AF) and BPH( benign prostate hyperplasia), Prostate Specific Antigen( PSA)

### ***C. esculenta*** protection against BPH is comparable to finasteride

In general *C. esculanta* fractions induced changes in serum total protein which were comparable to the standard treatment (finasteride) (Fig. 1). The mean serum total protein of normal controls (6.57 ± 0.56 mg/dl) were in the same range as *C. esculanta* treatments and significantly higher than the mean of testosterone propionate (TP) treatment group (6.18 ± 0.19 mg/dl). This was also observed in mean total body weight of the animals (Fig. 2B). The mean body weight of the rats in the normal control group was 166.8 ± 17.90 g which was significantly higher than in the TP group (127.76 ± 10.20 g). The mean weight of those treated with the various fractions were significantly higher than the TP group (TP + finasteride: 136.50 ± 32.80, TP + ECTECE: 154.60 ± 13.70, TP + HF: 136.30 ± 16.80, TP + DCMF: 137.06 ± 18.94, TP + BF: 142.60 ± 16.40, TP + EAF: 135.02 ± 16.90, TP + AF: 143.08 ± 8.90). Although the mean relative prostate weight (weight of each prostate divided by body weight of the animal) significantly (P < 0.05) increased by approximately 5 times (Fig. 2A) on treatment with testosterone propionate, this increase was moderated by *C.esculenta* extracts to levels comparable to that achieved by finasteride treatment.

### The effect of ethanol crude Tuber extract of ***c. esculenta*** and Fractions on the weights of vital organs

Administration of TP in male Wistar albino rats showed no significant (p > 0.05) difference on the relative liver, kidneys and heart weights although there was a significant (p < 0.05) reduction in mean relative testes weight by approximately 1.4 times less (Fig. 3(a-d)). Co-administration of TP and ethanol crude Tuber extract of *C. esculenta* (ECTECE), hexane fraction (HF), dichloromethane fraction (DCMF), butanone fraction (BF), ethyl acetate fraction(EAF) and aqueous fraction(AF) showed no significant (p < 0.05) difference on the relative organs weights (Fig. 3(a-d)).

### Effect of ethanol crude Tuber extract of ***c. esculenta*** and Fractions on Red blood cell and Platelet counts

Administration of TP in male Wistar albino rats showed no significant (p > 0.05) difference on RBC, haemoglobin, HCT, MCV, MCH, MCHC and platelets levels (Fig. 4(a-g)). Co-administration of TP and ethanol crude Tuber extract of *C. esculenta* (ECTECE), hexane fraction (HF), dichloromethane fraction (DCMF), butanone fraction (BF), Ethyl acetate fraction (EAF) and aqueous fraction(AF) in male rats showed no significant(p < 0.05) difference on the haematological parameters as shown in Fig. 4(a-g). A significant (p < 0.05) reduction in MCV level was recorded in the group that received TP + ECTECE while significant differences were recorded in groups that received TP + Finasteride and TP _+_ AF (Fig. 4g).

### Effect of ethanol crude Tuber extract of ***c. esculenta*** and Fractions on Red Blood cells and Leukocyte counts

Administration of TP in male Wistar albino rats significantly (p > 0.05) reduced the WBC, monocytes and lymphocytes counts with a significant (p < 00.05) elevation on the counts of neutrophils, eosinophil and basophiles (Fig. 5 (a-f)). Co-administration of TP and ethanol crude Tuber extract of *C. esculenta* (ECTECE), hexane fraction (HF), dichloromethane fraction (DCMF), butanone fraction (BF), Ethyl acetate fraction (EAF) and aqueous fraction (AF) in male rats significantly (p < 0.05) elevated the counts of WBC, monocytes and lymphocytes with significantly (p < 0.05) reduced neutrophil, eosinophil and basophil counts as shown in Fig. 5. There was no significant (p > 0.05) difference on WBC counts in group that received TP + ECTECE and non-significant (p > 0.05) difference on lymphocyte counts (Fig. 5(a-f)).

### Effect of ethanol crude Tuber extract of ***c. esculenta*** and Fractions on Histopathological features of the Prostate Gland

The results of histopathological examination of the prostate glands of the rats that were studied are shown in Fig. 6. Histopathological examination of the prostates of the control (Fig. 6 (I)) revealed no demonstrable prostatic hyperplasia and increased reduction in the number of cells that rimmed the prostatic gland with no proliferation of the glandular cells of the prostate. The group that received TP + finasteride group showed demonstrable prostatic hyperplasia as well as mild proliferation of the glandular cells of the prostate as shown in Fig. 6(II). The group that received TP + ECTECE (Fig. 6(III)) showed a mild proliferation of the glandular cells of the prostate and evidence of reduced production of prostatic gland fluids. There was also normal prostate gland and increased reduction of the number of cells that rimmed the prostate gland with increased eosinophilic secretions at the center. And finally, BPH untreated group showed increased proliferation, multiplication of the prostate gland and demonstrable hyperplasia (Fig. 6(IV)).

### Effect of ethanol crude Tuber extract of ***c. esculenta*** and Fractions on Histopathological features of liver

Liver histopathological examination showed no demonstrable features of hyperplasia, hypertrophy or atrophy in biopsies obtained from the normal control (Fig. 7 (I.NC)), BPH untreated group (Fig. 7(II.BPH)) as well as in the group that received TP + ECTECE (Plate 2 (III)). This demonstrated the safety of the extract to the liver. The only observed feature was congestion as shown in Fig. 7 (I-III).

### Effect of ethanol crude Tuber extract of ***c. esculenta*** and Fractions on Histopathological features of heart

Figure 8 (I-IV). Haematoxylin and Eosin stain on histological sections of heart tissue. (I) Normal control (NC) group (magnification, x200); (II) BPH control group (magnification, x200); (III) TP + Finasteride (magnification, x200) and (iv) TP + ECTECE (magnification, x200). BPH- Benign prostatic hyperplasia, ethanol crude tuber extract of *C. esculenta*.

### Effect of ethanol crude Tuber extract of ***c. esculenta*** and Fractions on Histopathological feature of kidneys

Histopathological examination of kidney tissue showed no demonstrable features of hyperplasia, hypertrophy or atrophy in biopsies obtained from the normal control, BPH control group, TP + Finasteride and TP + ECTECE as shown in Fig. 9 (I-IV). This demonstrated the safety of the extract to the kidneys.

## Discussion

This study was aimed at investigating the safety profile of *C. esculenta* in crude and semi purified form for the management and or prevention of BPH. Our results show that *C esculenta* is effective and widely tolerated as an anti BPH nutraceutical in the rat model. Although the exact natural products in *C. esculenta* that could explain its pharmacological actions have not been completely characterised, the methanol/chloroform extract has been reported to contain many bioactive compounds: hexadecanoic acid methyl ester, octadecanoic acid, 9,12-octadecadienoyl chloride, 11-octadecenoic acid methyl ester, 9-octadecenoic acid, 3-hexadecyloxycarbonyl-5-(2-hydroxylethyl)-4-methylimidazolium, hexanedioic acid, bis(2-ethylhexyl)ester and 3,5-di-t-butyl phenol. This is in addition to the high phenolic compounds content composed of Gallic Acid, Quercetin and Catechin [43, 44]. Many of these compounds have antioxidant (phenolic compounds), anti-inflammatory, anti-alopecic, 5-α-reductase inhibitory, anemiagenic, anti-tumor, immuno-stimulatory, anti-leucotriene-D_4_, anti-androgenic, lipoxygenase inhibitory and hypocholesterolemic properties ( organic compounds such as 9-octadecenoic acid ) [40].

Data from the biochemical and hematological parameters of our study do not show evidence of toxicity. However, we observed decrease in body weights of the treatment group. This is suggestive of a potential cellular response to arrest BPH through breakdown of tissue proteins [40]. In addition, we observed, an increase in the body weights of the groups that received finasteride, or ethanol crude tuber extract of *C. esculenta* (ECTECE) or hexane, dichloromethane, butanone, ethyl acetate and aqueous fractions of ECTECE which suggest bioactivity by the crude extract and fractions of ECTECE in halting the breakdown of tissue proteins.

More to this the elevated prostate weights as observed in the BPH untreated group could have resulted from pathological alterations in the prostatic tissue linked to BPH [40, 45]. However, the lower relative prostate weights of the BPH + finasteride, BPH + ECTECE, or BPH + hexane, dichloromethane, butanone, ethyl acetate and aqueous fractions of ECTECE groups indicate that these treatments may be able to reduce prostatic hyperplasia at the lower doses utilized in this investigation. Although we cannot rule out the possibility that increase in body weights of some rats contributed to the decrease in relative prostate weights, given that their bodyweights were much higher than those of the BPH untreated group.

According to the present study, non-significant (p > 0.05) differences in relative liver, kidney and heart weights were observed in both BPH untreated group and the treated groups. This non-significant difference in the relative’s organs weight may demonstrate nontoxic effect of the crude extract of cocoyam and fractions to the organs [46, 47]. This particular result confirms a previous report by Eleazu et al. (2013) [46] which showed no significant differences (𝑃 > 0.05) in the liver, kidney and heart weights of the diabetic rats administered cocoyam feed and diabetic control rats. Eleazu et al. (2014) [36] which reported that the diabetic rats fed with cocoyam had significant elevation (*P* < 0.05) of hepatic AST, ALT, ALP and serum proteins and albumin, but had significant reduction (*P* < 0.05) of blood glucose, serum urea, creatinine, amylase, lipase, AST, ALT and ALP compared with the diabetic control rats also support the safety of cocoyam in the management of diseases.

Reduction in the testes weight in the BPH untreated group as observed in this study could be as a result of exogenous effect of TP as a potential male contraceptive [48]. On the contrary the study revealed that crude extract cocoyam tuber and fractions were able to modulate the negative effect on the testes weight.

Increased counts of neutrophils, eosinophils, basophils and reduction in white blood cell, monocytes and lymphocytes counts; no significant (p > 0.05) difference on RBC, haemoglobin, HCT, MCV, MCH, MCHC and platelets counts could be attributed to the BPH induction in BPH untreated group. The treatment with crude extract of cocoyam tuber and fractions modulated the immune and haematological parameters showing the safety of the cocoyam extract and fractions. This result is similar with Princewill-Ogbonna et al. (2016) [49] which reported non-significant difference in haematological parameters in rats feed with cocoyam made feed. Azubuike et al. (2018) [47] also reported the safety of usage of C. *esculenta* leaves in the reduction of fat adipose tissues and its ameliorative effect on HFD-induced liver damage.

## Conclusion and recommendation

Our results suggest that *C.esculenta* has a potential for use as a nutraceutical in benign hyperplasia thus we recommend further studies on mechanism and the active components present. Given that *C.esculenta* is a commonly consumed food in many African countries it would be nice to do epidemiological studies in these areas with higher consumption of this important food. And probably be subjected to clinical trials aimed complementing standard therapies as a nutraceutical.

## Data Availability

All data generated or analyzed during this study are included in this published article.
